# Regulation of foamy virus protease activity by viral RNA

**DOI:** 10.1186/1742-4690-8-S1-A228

**Published:** 2011-06-06

**Authors:** Maximilian J Hartl, Jochen Bodem, Fabian Jochheim, Axel Rethwilm, Paul Rösch, Birgitta M Wöhrl

**Affiliations:** 1 Lehrstuhl Biopolymere, Universität Bayreuth, Bayreuth, 95447, Germany; 2 Institut für Virologie und Immunbiologie, Würzburg, 97978, Germany

## 

Foamy viruses synthesize the Pol precursor protein from a specific transcript. Thus, in contrast to orthoretroviruses, e.g. human immunodeficiency virus, no Gag-Pol precursor protein is synthesized. Foamy viral Pol is processed into a mature protease-reverse transcriptase fusion protein (PR-RT) and the integrase. To avoid premature processing of the Gag and Pol precursors the activity of the protease domain needs to be inhibited before virus assembly. We have demonstrated recently that an independent foamy virus protease domain as well as the PR-RT fusion protein are monomers lacking protease activity. We thus postulated protease activation through induction of dimerization by an additional factor. Here, we identify a specific protease activating RNA motif (PARM) located in the pol region of viral RNA which stimulates foamy virus protease activity in vitro and in vivo, revealing a novel and unique mechanism of retroviral protease activation. This mechanism is strikingly different to that of orthoretroviruses, where the protease can be activated even in the absence of viral RNA during the assembly of virus-like particles. Although it has been shown that the integrase domain is important for Pol uptake, activation of the foamy virus protease is integrase independent. We show that only RNAs containing the PARM result in significant activation of the protease. DNA harboring the PARM is not capable of protease activation. Structure determination of the PARM by selective 2’ hydroxyl acylation analyzed by primer extension (SHAPE) revealed a distinct RNA folding, important for protease activation and thus virus maturation.

